# What is the optimal length of an Online Forward Triage Tool? The need for a framework

**DOI:** 10.7189/jogh.10.0203103

**Published:** 2020-12

**Authors:** Janet Michel, Annette Mettler, Wolf E Hautz, Thomas C Sauter

**Affiliations:** 1 Department of Emergency Medicine, Inselspital University Hospital, University of Berne, Berne, Switzerland

The COVID-19 pandemic has spurred a boom of online digital tools. Online symptom checkers and similar tools like chatbot, have the potential to get patients to appropriate care provider, improve satisfaction, outcomes and even shift cost curves downwards [1]. If done properly, online forward triage tools (OFTTs) can facilitate meaningful patient engagement [1]. As countries impose lock down restrictions, more people are engaging with symptom checkers and other online tools. According to a poll, non-related to the pandemic, 75% of respondents revealed that they research their condition before a visit to hospital [1].

During this COVID-19 pandemic, telemedicine has emerged as an effective and practical solution in prevention, treatment and reduction of the spread of COVID-19 [2]. Telemedicine refers to communication and information technologies used to overcome physical and geographical barriers thereby increasing access to health care services [3]. Examples of telemedicine in use are: online self-assessment of symptoms by patients before being seen by health care provider, chronic patients having remote consultations and medicine delivered home, to minimize risk of exposure and quarantined health care providers who can continue to see patients through video consultations [2]. According to a recent survey, 84% of the patients revealed that they are likely to select a health care provider that offers telemedicine compared to those that do not-patients seem to be embracing telemedicine [2].

## SOME OFTT CHALLENGES

Building deep learning systems does not only depend on quantity of data used but type and quality. The challenge is that time- years are needed to test and fine tune tools with thousands of cases for them to continue learning. The process is unfortunately not automatic, requiring human intervention and oversight [4]. Similar online tools, expert systems were used in the 80s in aviation, finance and medicine including MYCIN, a tool that advised which antibiotic to use for which infection [5,6]. Another glaring challenge is tool accuracy, which depends on foundations of the clinical engine, a challenge that expert systems also confronted [6]. Organizations providing online tools to patients should pay attention to the tools` clinical engine foundations. Untested and unvalidated clinical engines tend to give erroneous information and recommendations [1]. How many online forward triage tools (OFTTs) in use have been peer reviewed for accuracy is not clear? Engines that are vetted medically, should be able to meet all individual consumer experiences, incorporating both self-diagnosis and triaging patients and directing them to the appropriate level of care. One study of online tools revealed deficits in both diagnosis and triage, with triage advice being risk averse- recommending medical care to patients who could have done with self-care [7].

The following are suggested as questions that need to be answered before providing online services [1]:

1. Is the engine validated medically by an independent source?
2. How many published studies have peer reviewed the engine?
3. How many conditions are covered by the engine?
4. How wide is the range of symptoms covered by the engine?
5. Is the application programming interface (API) published and is the tool incorporated into user experience and workflow platforms.

Another challenge of OFTTs is that the pandemic linchpin lies in population-wide testing, a service telemedicine cannot offer currently [2]. The following additional challenges were revealed in a symptom checker systematic review:

- Evidence revealed weaknesses in patient safety,
- There were variations in accuracy of diagnosis which was also low
- The algorithm for triage was more averse to risk as compared to health care workers
- evidence of patients compliance showed that the younger and highly educated tended to use OFTTs, pointing at equity issues [8]

## A GAP

Consumers seem to want the best possible medical and health information and seem to value the same highly tested information tools made available to care providers, but presented in simpler terms [1]. One poll revealed that 86% of respondent said the more information including symptoms, they supplied about their condition, the more appropriate the treatment they will get from their health care provider [1]. What is however not clear, is the type of patients polled. Were these patients with chronic conditions like chronic pain, or were these patients who had an acute condition eg, shortness of breath and needed to know what to do? In case of the latter scenario, we are of the mind that those with an acute condition would most probably be in a physically and cognitively compromised state, to self-assess using an OFTT.

**Figure:**
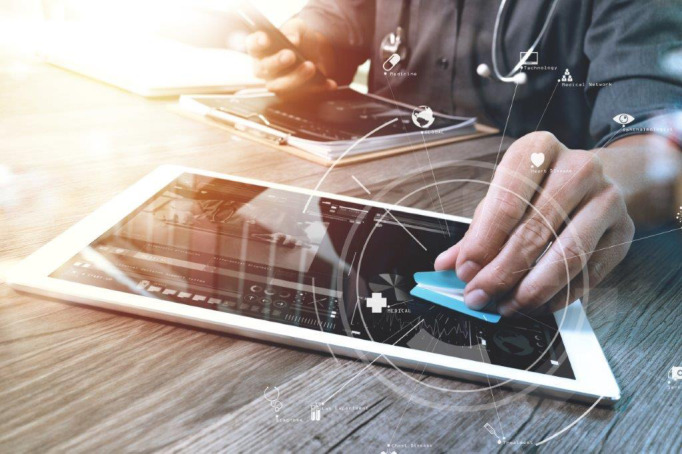
Photo: Purchased by the authors from canstockphoto.

Should COVID-19 OFTTs, an acute condition, a pandemic situation be different from a general OFTT such as one for chronic pain? We would like to suggest-yes. How much time, on average should patients spend filling these in? This again, we would like to say depends on the condition; acute vs chronic. What is the optimal length of an OFTT questionnaire, how many questions should be asked, average, minimum, maximum? What is the optimal time a patient should spend filling in responses? What is the maximum acceptable time, a patient should spend filling OFTT responses, before being told to call 911? The purpose of this paper is to stimulate a discussion and hopefully generate answers to the above questions. One paper proposes questions to ask when setting up an OFTT [4], but does not give the average number of questions, average time nor do they differentiate between acute and chronic conditions.

## TOWARDS A FRAMEWORK FOR OFTTS

We suggest a differentiation between OFTTs for chronic vs those for acute conditions. A patient with chronic pain might have become accustomed to the pain, and might find that giving all the particular differences of pain intensity and duration and triggers etc, will make the doctor better understand their pain. A patient, who on the other hand is in an acute state, has severe symptoms, is distressed and wants to know what to do next, might be discouraged or be in physical danger if asked to respond to 50 questions, until he gets a recommendation to call 911. In acute conditions time is critical in several ways;

- Time to fill in responses: very ill and distressed patients might have difficulty to sit and concentrate for longer than 5 minutes, particularly if dyspnoeic, vomiting or in severe pain.
- Time as a determinant factor in the prognosis: in conditions such as myocardial infarction, time becomes a determinant factor in the prognosis. A 30-minute delay in presenting to emergency room (ER) can make a difference, hence the need to reduce the OFTT time in cases such as these.

We are therefore of the opinion that there should be cases, in which patients should be advised to discontinue the OFTT and get immediate emergency rescue services, lest they lose precious time. For some acute conditions like myocardial infarction, we are of the mind that such OFTTs, in fact be discouraged.

## Future studies

Do OFTTS have room in all fields of Medicine? The value of OFTTs in chronic disease, screening, diagnosis and follow-up has been demonstrated [4]. The role of OFTTs in paediatrics, neurology and acute settings are still contested, as states often change suddenly. More studies are needed in this regard. As OFTT use increases, we propose the following research questions: How many questions are optimal or how much time is optimal, for patients, to spend filling an OFTT in acute and chronic cases? Our hypothesis is that the longer the time spent on filling in OFTT responses and the higher the number of questions, the lower the questionnaire completion rates, the lower the proportion of recommendations followed and consequently poor outcomes. We also propose the assessment of COVID-19 OFTTs in Switzerland, comparing the number of questions, the average length of time spent filling in OFTT responses and outcomes like questionnaire completion rates and proportion of recommendations followed.

## CONCLUSION

As OFTT use increases, their optimal lengths have not been explicitly defined. We therefore propose that frameworks for OFTT evaluation be updated to include among others, dropout rates, optimal number of questions and time spent filling in OFTT responses, validation status of clinical engines along with accuracy of diagnosis and triage.
